# Enhancing chimeric antigen receptor T‐cell immunotherapy against cancer using a nanoemulsion‐based vaccine targeting cross‐presenting dendritic cells

**DOI:** 10.1002/cti2.1157

**Published:** 2020-07-22

**Authors:** Jack D Chan, Bianca von Scheidt, Bijun Zeng, Amanda J Oliver, Ashleigh S Davey, Aesha I Ali, Ranjeny Thomas, Joseph A Trapani, Phillip K Darcy, Michael H Kershaw, Riccardo Dolcetti, Clare Y Slaney

**Affiliations:** ^1^ Cancer Immunology Program Peter MacCallum Cancer Center Melbourne VIC Australia; ^2^ Sir Peter MacCallum Department of Oncology University of Melbourne Parkville VIC Australia; ^3^ The University of Queensland Diamantina Institute Translational Research Institute Woolloongabba QLD Australia

**Keywords:** CAR T cells, Clec9A, cross‐presentation, dendritic cells, nanoemulsion, vaccine

## Abstract

**Objectives:**

Adoptive transfer of chimeric antigen receptor (CAR)‐modified T cells is a form of cancer immunotherapy that has achieved remarkable efficacy in patients with some haematological cancers. However, challenges remain in CAR T‐cell treatment of solid tumours because of tumour‐mediated immunosuppression.

**Methods:**

We have demonstrated that CAR T‐cell stimulation through T‐cell receptors (TCRs) *in vivo* can generate durable responses against solid tumours in a variety of murine models. Since Clec9A‐targeting tailored nanoemulsion (Clec9A‐TNE) vaccine enhances antitumour immune responses through selective activation of Clec9A^+^ cross‐presenting dendritic cells (DCs), we hypothesised that Clec9A‐TNE could prime DCs for antigen presentation to CAR T cells through TCRs and thus improve CAR T‐cell responses against solid tumours. To test this hypothesis, we used CAR T cells expressing transgenic TCRs specific for ovalbumin (OVA) peptides SIINFEKL (CAROTI) or OVA_323‐339_ (CAROTII).

**Results:**

We demonstrated that the Clec9A‐TNEs encapsulating full‐length recombinant OVA protein (OVA‐Clec9A‐TNE) improved CAROT T‐cell proliferation and inflammatory cytokine secretion *in vitro*. Combined treatment using the OVA‐Clec9A‐TNE and CAROT cells resulted in durable responses and some rejections of tumours in immunocompetent mice. Tumour regression was accompanied by enhanced CAROT cell proliferation and infiltration into the tumours.

**Conclusion:**

Our study presents Clec9A‐TNE as a prospective avenue to enhance CAR T‐cell efficacy for solid cancers.

## Graphical abstract

Clec9A‐targeting tailored nanoemulsion (Clec9A‐TNE) induced extensive proliferation, persistence and activation of chimeric antigen receptor (CAR) T cells, leading to the eradication of solid tumours in murine models. This novel approach could lead to the development of new CAR T‐cell therapies against some common solid tumour types in patients.

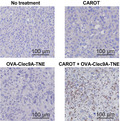

## Introduction

Chimeric antigen receptor (CAR) T‐cell treatment against blood cancers has been promising, demonstrating significant responses in patients with certain B‐cell malignancies.^1^, ^2^, ^3^ The success of CAR T‐cell treatment has resulted in FDA approvals for two CAR T‐cell therapies, including CD19 CAR T‐cell therapies for the treatment of children and adolescents with acute lymphoblastic leukaemia (ALL) and adults with relapsed‐refractory large B‐cell lymphoma in 2017. Despite the successes in treating these haematological cancers, clinical trials targeting solid tumours have demonstrated a mostly inadequate efficacy. This is largely attributed to the immunosuppressive tumour microenvironment (TME) that inhibits T‐cell function, and to the poor trafficking of CAR T cells to the tumour sites.^4^, ^5^

In a previous study, we utilised ‘CARaMEL’ dual‐specific T cells, expressing a CAR specific for Her2 tumour antigen, and a T‐cell receptor (TCR) specific for a strong, tumour‐unrelated immunogen, the melanocyte protein gp100 (also known as pMEL). When the CARaMEL T cells were used in combination with a live vaccinia viral vaccine encoding gp100 (VV‐gp100), the treatment induced dramatic CAR T‐cell expansion *in vivo* through the stimulation of gp100 TCR and resulted in the eradication of large tumours and metastases in a number of murine Her2 tumour models.^6^ Despite the successes of this dual‐specific CAR T‐cell approach, it would be ideal to develop a more convenient and readily approvable vaccine that does not involve a live virus.

Recently, we used a tailored nanoemulsion (TNE) to encapsulate tumour antigens for the presentation to the cross‐presenting dendritic cells (DC) through the specific receptor Clec9A (Clec9A‐TNE).^7^ Clec9A recognises the necrotic cell ligand F‐actin for the uptake of cell‐derived antigens, which are processed for MHC class I and class II presentation.^8^ When OVA protein was encapsulated into Clec9A‐TNE (OVA‐Clec9A‐TNE), it demonstrated effective targeting and activation of cross‐presenting DCs, even in the absence of additional adjuvant, and generated greater OVA‐specific T‐cell expansion than antibody‐based targeting of OVA to Clec9A^+^ DCs in mice. Effective priming of OVA‐specific T cells significantly delayed tumour growth and improved survival in mice bearing orthotopic OVA‐expressing tumours. This effect also extended to Clec9A‐TNEs carrying B16F10 tumour neo‐epitopes. These TNEs elicited epitope‐specific T‐cell responses that significantly delayed tumour growth and enhanced the survival of tumour‐bearing mice in an epitope‐specific manner.^7^

In the current study, we hypothesised that Clec9A‐TNE could prime DCs for antigen presentation to CAR T cells through TCRs and thus improve CAR T‐cell responses against solid tumours. We used the dual‐specific CAR T cells expressing a second‐generation anti‐Her2 CAR (CD3‐ζ/CD28), and OTI or OTII TCRs (CAROTI TCR specific to the OVA peptide SIINFEKL or CAROTII TCR specific to OVA_323‐339_).^9^, ^10^ Thus, the CAROT cells have dual specificity for Her2 and OVA peptide antigens through their CARs and TCRs, respectively. We aimed to investigate whether the OVA‐Clec9A‐TNE could facilitate antigen presentation to the TCRs of CAROT cells, thereby enhancing CAROT cell activation *in vitro* and antitumour responses *in vivo*.

## Results

### Dual‐specific CAROTI and CAROTII cells express CAR and OVA‐specific TCRs

**Figure 1:**
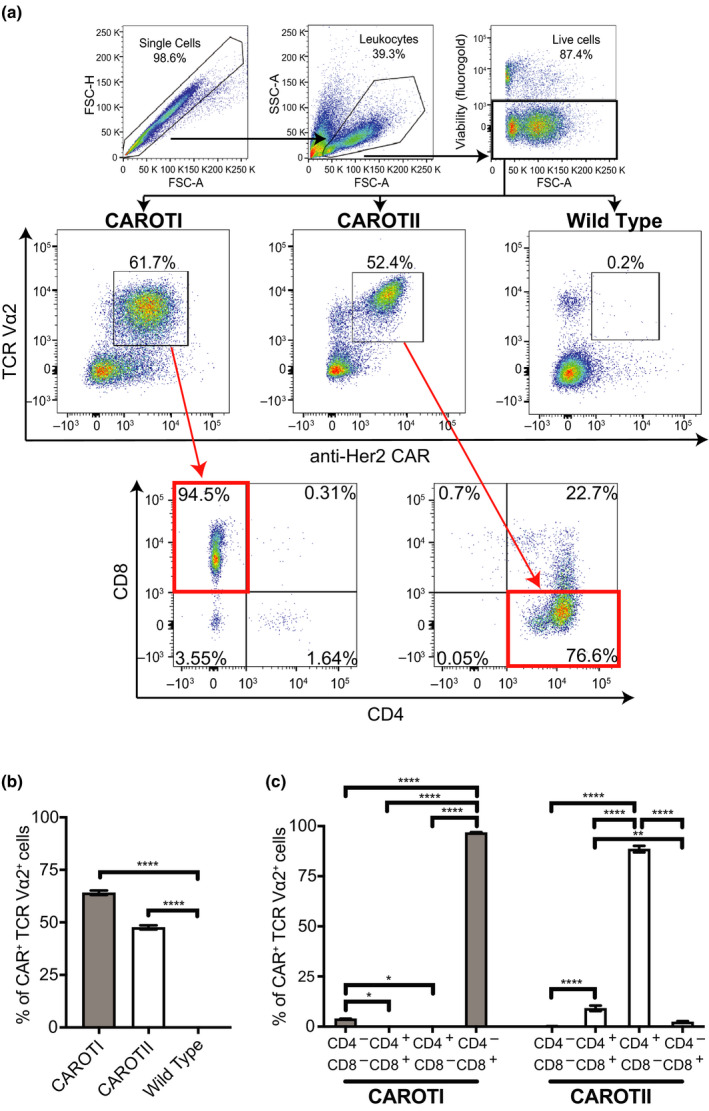
CAROTI and CAROTII expressing anti‐Her2 CAR and TCR Vα2. Splenocytes isolated from CAROTI, CAROTII and WT mice were stained with a viability marker (fluorogold) and antibodies against CD4, CD8, TCR Vα2 and c‐Myc tag (CAR). Cells were phenotypically analysed by flow cytometry for CD4, CD8, anti‐Her2 CAR and TCR Vα2 expression on viable cells. **(a)** Gating strategy and representative plots of CAROTI and CAROTII cells co‐expressing the anti‐Her2 CAR and TCR Vα2 of two independent experiments. **(b)** Anti‐Her2 CAR and TCR Vα2 co‐expression as a percentage of total lymphocytes. **(c)** CAROTI and CAROTII CD4 and CD8 percentages of dual‐specific CAROT cells. **(b, c)** Data are represented as the mean ± the standard error of the mean (SEM) and are pooled from two independent experiments in triplicates. Statistical significance was determined by one‐way ANOVA. *P < 0.05, **P < 0.01, ****P < 0.0001.

In this study, we crossed C57BL/6 Her2‐CAR transgenic mice to the OTI or OTII mice.^9^, ^10^ The cells from the Her2‐CAR mice express a second‐generation CAR (anti‐Her2‐CD28‐CD3ζ) driven by a vav promoter.^11^ Thus, all of the T cells from the resulting CAROT mice express both a CAR specific for Her2 and a TCR specific for OVA, derived either from OTI for CD8 T cells or OTII for CD4 T cells. We refer to these mice and T cells as CAROTI^12^ and CAROTII. To confirm co‐expression of the CAR and TCR, splenocytes were harvested from CAROTI and CAROTII mice and stained with fluorochrome‐conjugated antibodies specific for a c‐Myc tag included in the CAR construct, and the TCR Vα2 chain that comprises OTI and OTII TCRs (Figure 1a). By using the gating strategy detailed in Figure 1a, we found simultaneous, high‐level CAR and TCR Vα2 expression on CAROTI and CAROTII T cells (Figure 1b). The dual‐specific CAROTI cells were mostly CD8^+^, while dual‐specific CAROTII cells were mostly CD4^+^ (Figure 1c).

### CAROT T cells respond through their TCR and CAR

**Figure 2:**
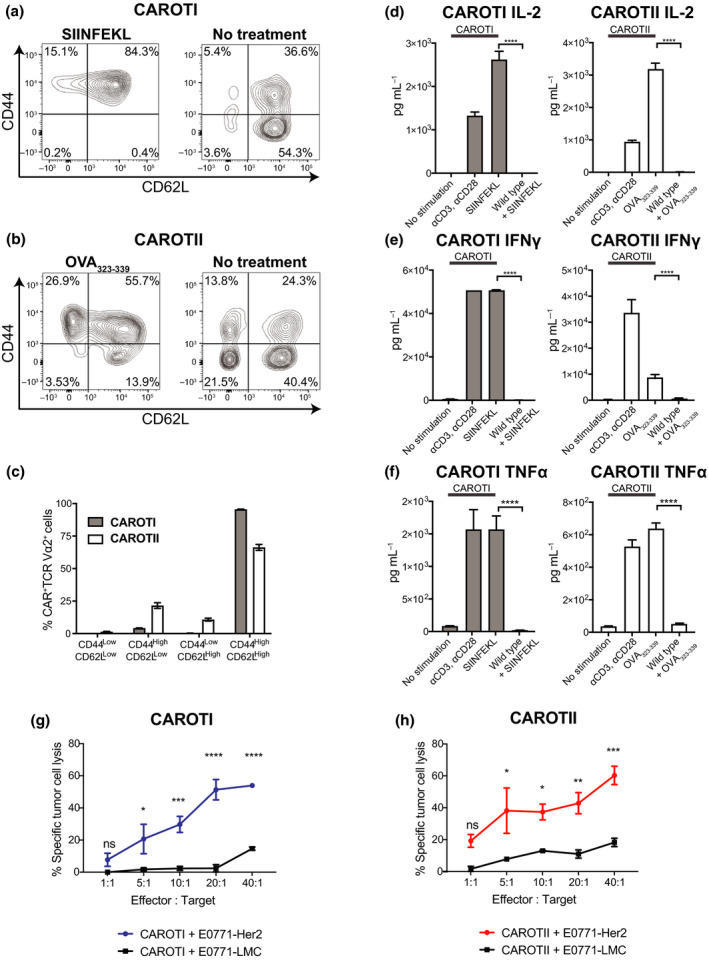
CAROTI and CAROTII cells express functional transgenic TCRs and CARs. CAROTI or II splenocytes were stimulated with 5 μm SIINFEKL or OVA_323‐339_, respectively, over 48 h. WT splenocytes were used as the experimental control. Splenocytes cultured in medium (no stimulation) or αCD3 (0.5 μg mL^−1^) and αCD28 (0.5 μg mL^−1^) were used as negative and positive controls, respectively. Cells were analysed via flow cytometry and cultured with ^51^Cr labelled E0771‐Her2 or E0771‐LMC tumour cells to test CAROT cell cytotoxicity. Supernatants were harvested and analysed for cytokine secretion. **(a, b)** Representative CD44 and CD62L expression of CAROT cells. **(c)** Percentages of CAROTI and CAROTII cells expressing CD44 and CD62L after stimulated with 5 μm SIINFEKL or OVA_323‐339_, respectively, over 48 h. **(d)** IL‐2, **(e)** IFN*‐*γ and **(f)** TNF*‐*α levels in the cell culture supernatant. **(g, h)** Chromium release assay of CAROT cells against Her2^+^ (E0771‐Her2) and Her2^−^ (E0771‐LMC) cancer targets. Data are presented as mean ± SEM from three independent experiments in triplicates. Significance was calculated using one‐way ANOVA. *P < 0.05, **P < 0.01, ***P < 0.001, ****P < 0.0001, ns = not significant.

We next investigated whether TCRs and CAR on the CAROT T cells could respond to cognate peptide antigens. To test TCR function, CAROTI and CAROTII splenocytes were incubated with peptides cognate for their corresponding OTI and OTII TCRs (SIINFEKL and OVA_323‐339_, respectively). The CAROTI and CAROTII splenocytes and culture supernatants were then harvested for flow cytometric and cytometric bead assay (CBA) analysis. Based on expression levels of CD44 and CD62L, CAROT cells were classified as CD44^Low^CD62L^High^, CD44^High^CD62L^High^, CD44^High^CD62L^Low^ and CD44^Low^CD62L^Low^ populations. In response to cognate peptide antigens, both CAROTI and CAROTII cells were predominantly CD44^High^CD62L^High^ (Figure 2a–c).

When cultured with their respective cognate peptides, CAROTI and CAROTII cells secreted IL‐2, IFN‐γ and TNF‐α (Figure 2d–f), confirming that CAROT cells express functional OTI/OTII TCRs and that stimulation through the TCRs could activate CAROT cells.

To confirm the functional capacity of the anti‐Her2 CAR, we determined whether CAROT cells could kill Her2^+^ tumour cells in an antigen‐dependent manner. Uniformly high Her2 expression was confirmed on E0771‐Her2 tumour cells, using flow cytometry (Supplementary figure 1). After activation with their respective cognate peptide antigens for 7 days, CAROTI and CAROTII cells mediated strong Her2‐specific cytolysis of tumour cells (Figure 2g and h).

In summary, CAROTI and CAROTII cells have a functional anti‐Her2 CAR that can mediate Her2‐specific killing and OVA‐specific TCRs that can activate the CAROT T cells.

### OVA‐Clec9A‐TNE drives CAROTI and CAROTII cell activation

**Figure 3:**
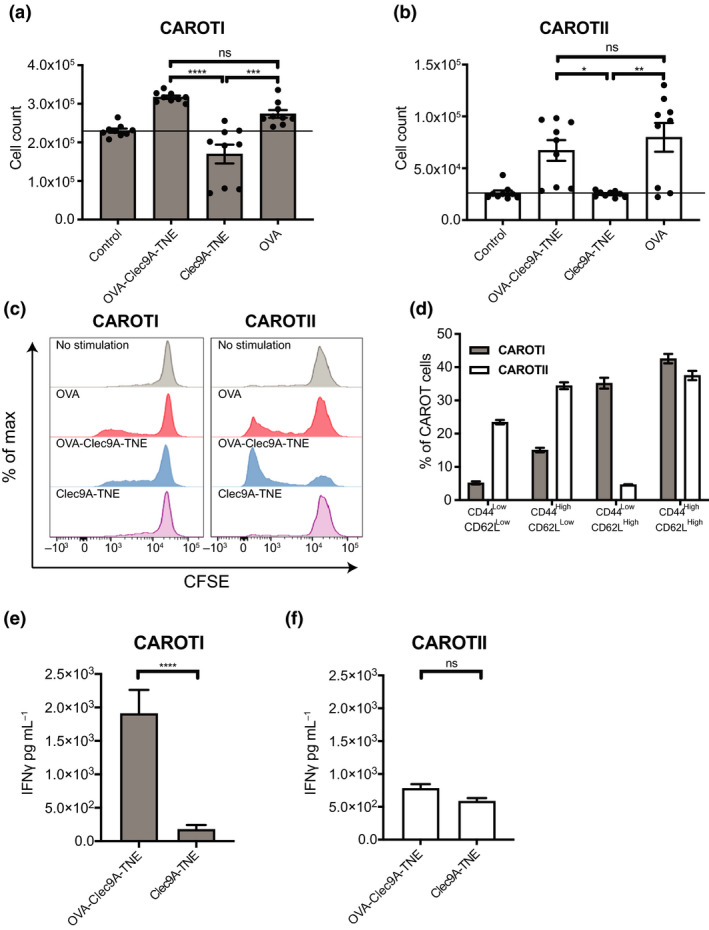
OVA‐Clec9A‐TNE drives CAROT cell proliferation and IFN*‐*γ secretion. 1 × 10^6^ CAROTI and CAROTII splenocytes labelled with 0.1 μm CFSE (**b**) or without CFSE (**a**, **c**–**f**) were cultured over 72 and 96 h, respectively, with full‐length OVA protein or OVA‐Clec9A‐TNE at an OVA concentration of 50 nm. Splenocytes were cultured with an empty‐vehicle TNE at dilutions corresponding to the amount of TNE added in OVA‐Clec9A‐TNE cultures. Splenocytes cultured with media (no stimulation, represented with a horizontal bar where appropriate) or with soluble αCD3 (0.5 μg mL^−1^) and αCD28 (0.5 μg mL^−1^) were used as negative and positive controls, respectively. Following culture, CAROT cells were quantified via flow cytometric analysis and the amount of IFN*‐*γ secreted into supernatants was measured. **(a)** CAROTI and **(b)** CAROTII cell counts in the splenocyte culture. **(c)** Representative histograms of CAROTI and CAROTII cell proliferation in CFSE dilution. **(d)** Percentages of CAROTI and CAROTII cells showing high or low CD44 and CD62L expression following culture with OVA‐Clec9A‐TNE at a 50 nm OVA concentration. **(e)** CAROTI and **(f)** CAROTII splenocyte culture IFN*‐*γ secretion. **(a, b, d, e, f)** Data are presented as mean ± SEM pooled from three independent experiments in triplicates. Statistical significance was determined using one‐way ANOVA (*P < 0.05, **P < 0.01, ***P < 0.001, ****P < 0.0001, ns = not significant).

To evaluate whether OVA‐Clec9A‐TNE can activate CAROT cells, we stimulated the cells with 50 nm full‐length OVA protein or the OVA‐Clec9A‐TNE *in vitro*. An empty‐vehicle Clec9A‐TNE was used to assess any non‐specific effects that the nanoemulsion may produce. OVA‐Clec9A‐TNE stimulated CAROTI and CAROTII cell proliferation comparably to OVA protein, as was reflected both by cell counts (Figure 3a and b) and CFSE dilution (Figure 3c). The empty‐vehicle Clec9A‐TNE caused no proliferation above the medium only control.

After incubation with the OVA‐Clec9A‐TNE, a significant proportion of CAROTI and CAROTII cells were phenotypically CD44^High^CD62L^High^ (Figure 3d), as occurred when the cells were stimulated with their cognate peptides (Figure 2c). Interestingly, CAROTI cells included a significant proportion of CD44^Low^CD62L^High^ cells signifying naïve or stem cell memory‐like phenotype, whereas CAROTII cells had a greater percentage of CD62L^Low^CD44^High^ cells, characteristic of an effector phenotype. We further analysed the CFSE labelled CAROTI cells presented in Figure 3c and found that the majority of the CD44^Low^CD62L^High^ cells were CFSE^High^ (Supplementary figure 2), indicating that these cells were naïve and had not been stimulated. CAROTI, but not CAROTII cells, secreted significantly higher levels of IFN‐γ when cultured with OVA‐Clec9A‐TNE than when cultured with TNE vehicle control (Figure 3e and f).

### OVA‐Clec9A‐TNE augments CAROT cell antitumour function *in vivo*

We have demonstrated that TCR stimulation of dual‐specific T cells with a live virus vaccine can drive durable, complete remission of a variety of Her2 expressing tumours *in vivo*.^6^, ^13^ To maximise the opportunity for clinical translation, we now aim to achieve similar outcomes using a more generic and clinically tractable approach that does not require administering a live virus. To this end, we used immunocompetent, transgenic C57BL/6‐Her2 mice that are fully tolerant of, and constitutively express human Her2 in breast epithelium and cerebellar cortex, and are also permissive for the growth of Her2 expressing tumours of C57BL/6 origin.^14^, ^15^

**Figure 4:**
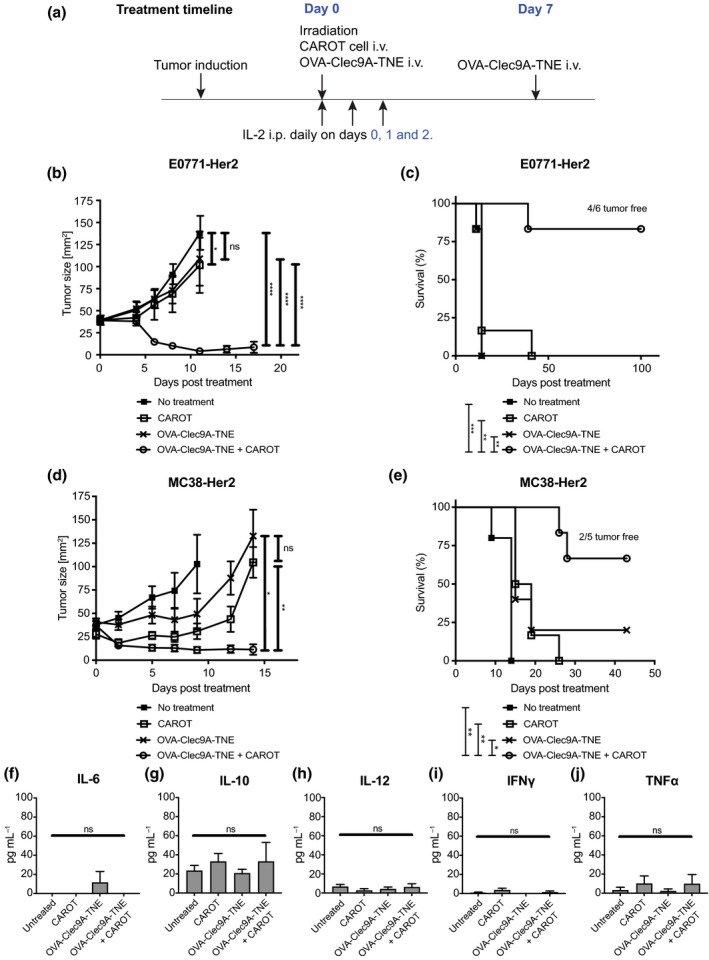
OVA‐Clec9A‐TNE and CAROT cell combination therapy mediates tumour regression and prolonged survival. C57BL/6 human‐Her2 transgenic mice were injected with 5 × 10^5^ E0771‐Her2 or MC38‐Her2 tumour cells subcutaneously. After 8 days (day 0), tumour‐bearing mice were left untreated or received a preconditioning dose of 4 Gy total body irradiation. Irradiated mice received intravenous (i.v.) injections of 1 × 10^7^ CAROTI cells and 1 × 10^7^ CAROTII cells, OVA‐Clec9A‐TNE (10 μg 100 μL^−1^) or a combination of CAROTI cells, CAROTII cells, and OVA‐Clec9A‐TNE. Treated mice received intraperitoneal (i.p.) injections of IL‐2 (1 × 10^5^ IU 100 μL^−1^) on days 0, 1 and 2 post*‐*CAROT cell transfer. Mice that received OVA‐Clec9A‐TNE also received an additional injection of OVA‐Clec9A‐TNE on day 7. **(a)** Treatment timeline. **(b, d)** Tumour growth curve. Statistical significance was determined by two‐way ANOVA. **(c, e)** Survival curves of treated and untreated mice. Statistical significance was determined by the Mantel–Cox test. Quantification of **(f)** IL‐6, **(g)** IL‐10, **(h)** IL‐12, **(i)** IFN*‐*γ and **(j)** TNF*‐*α from sera of mice bearing E0771‐Her2 tumours. Statistical significance was determined by one‐way ANOVA (*P < 0.05, **P < 0.01, ***P < 0.001, ****P < 0.0001, ns = not significant). Data are shown as mean ± SEM of 5 or 6 mice per group from one experiment per tumour model.

In the Her2 transgenic mice bearing E0771‐Her2 breast cancers, we observed significant tumour growth inhibition in mice receiving both adoptively transferred CAROT cells (CAROTI+CAROTII) and injections of OVA‐Clec9A‐TNE (the treatment scheme is described in Figure 4a). In contrast, CAROT cell‐ or OVA‐Clec9A‐TNE treatment alone had a lesser antitumour effect (Figure 4b). In fact, tumours in some mice that received the combination therapy regressed completely, leading to long‐term survival (Figure 4c). In contrast, none of the mice in the no treatment group or the OVA‐Clec9A‐TNE‐treated group survived beyond day 14. Similar results were obtained in Her2‐transgenic C57BL/6 mice bearing MC38‐Her2 tumours. Significant tumour growth inhibition (Figure 4d) and prolonged survival (Figure 4e) were observed in mice receiving CAROT cells and OVA‐Clec9A‐TNE in combination.

We did not observe overt signs of toxicity such as breast or cerebellar inflammation in the treated mice. In addition, to assess potential cytokine hypersecretion associated with the treatment, serum cytokine levels were measured. The pro‐inflammatory cytokines IL‐12, IFN‐γ and TNF‐α, the pleiotropic cytokine IL‐6 and the immunosuppressive cytokine IL‐10 were not significantly elevated in any treatment group (Figure 4f–j).

### Combined OVA‐Clec9A‐TNE and CAROT treatment generates an antitumour memory response in the long‐term surviving mice

**Figure 5:**
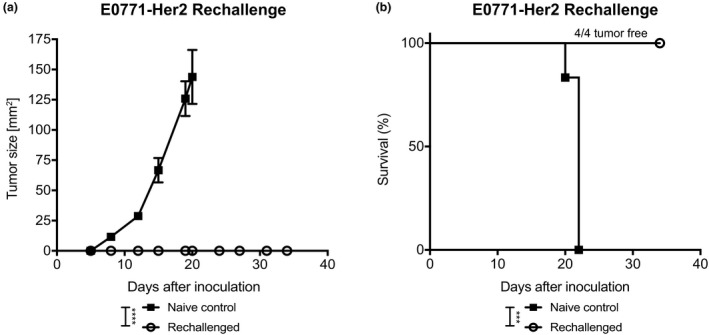
Surviving OVA‐Clec9A‐TNE and CAROT cell*‐*treated mice are resistant to tumour cell rechallenge. Surviving C57BL/6 human‐Her2 transgenic mice that rejected E0771‐Her2 tumours following OVA‐Clec9A‐TNE and CAROT cell combination treatment (*n* = 4) and naïve control mice (*n* = 6) were challenged with 1 × 10^5^ E0771‐Her2 cells via subcutaneous injection on the opposite flank to the primary tumour challenge. **(a)** Tumour growth curve of naïve control and rechallenged mice. Data are shown as mean ± SEM. Statistical significance was determined by two‐way ANOVA. **(b)** Survival curve of naïve control and rechallenged mice. Statistical significance was determined by the Mantel–Cox test for survival (***P < 0.001, ****P < 0.0001). Results are from one experiment.

An important aim of immunotherapy is to generate immune memory to prevent tumour relapse. Therefore, we investigated whether combining OVA‐Clec9A‐TNE and CAROT cells could produce long‐term protection. Mice from the OVA‐Clec9A‐TNE and CAROT treatment group that had completely rejected their tumour were rechallenged with a further subcutaneous inoculation of E0771‐Her2 tumour cells in the opposite flank after remaining tumour‐free for 60 days. We observed no tumour growth in rechallenged mice, while tumours grew progressively in naïve control mice (Figure 5a). All rechallenged mice achieved prolonged survival, while naïve control mice did not survive beyond 22 days after tumour cell challenge (Figure 5b), confirming that the combined therapy induced an antitumour memory response.

### OVA‐Clec9A‐TNE drives CAROTI cell expansion and infiltration into the tumours

**Figure 6:**
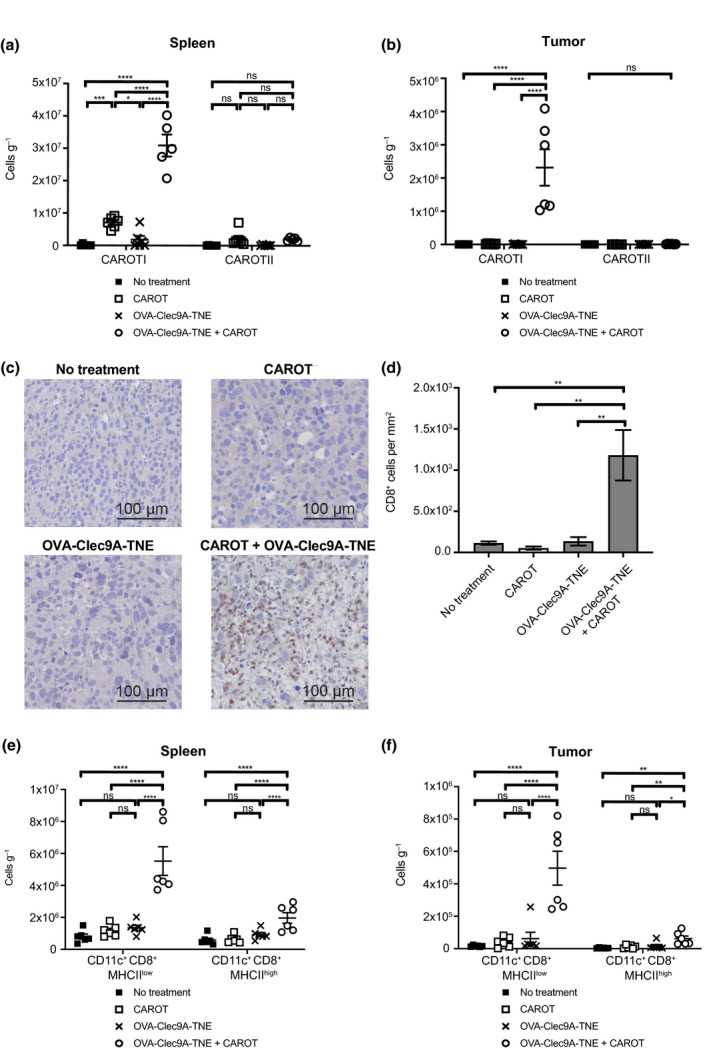
OVA‐Clec9A‐TNE promotes CAROTI expansion and tumour infiltration. E0771‐Her2 tumour‐bearing C57BL/6‐Her2 transgenic mice were treated as shown in Figure 4a. Spleens and tumours were harvested 5 days after the treatments. Single‐cell suspensions were stained for CAROT cell congenic marker expression (CD45.1) or the DC markers CD11c, CD8 and MHC Class II. CAROT cell numbers in the **(a)** spleens and **(b)** tumours. **(c)** Representative IHC images showing CD8^+^ staining (brown) in the tumour tissue. **(d)** Whole IHC section CD8^+^ cell counts from three random sections per group enumerated using HALO software shown as mean ± SEM. **(e)** The number of CD11c^+^CD8^+^ DCs expressing MHCII^low^ or MHCII^high^ per gram of tissue in the spleens and tumours. **(a, b, e, f)** Data are shown as mean ± SEM of 5 or 6 mice per group and are representative of two independent experiments. Statistical significance was determined by one‐way ANOVA (*P < 0.05, **P < 0.01, ***P < 0.001, ****P < 0.0001, ns = not significant).

Spleens and tumours from tumour‐bearing mice were analysed 5 days post‐therapy. We observed significantly higher CAROTI cell numbers in the spleens and tumours of mice administered OVA‐Clec9A‐TNE and CAROT cell adoptive transfer (Figure 6a and b) compared to the other treatment groups. Surprisingly, there was no significant increase in CAROTII cells in the spleens or tumours of mice that received combination therapy compared to untreated mice or mice that received monotherapies. IHC analysis demonstrated a dense tumour infiltration of CD8^+^ T cells in combination‐treated mice, and the CD8^+^ T cells were evenly distributed in the tumour tissue (Figure 6c and d).

Analysis of DC markers revealed a higher proportion of cross‐presenting CD11c^+^CD8^+^MHCII^Low^ and CD11c^+^CD8^+^MHCII^High^ DCs in the spleens and tumours of mice administered combination therapy (Figure 6e and f) when compared to mice in other groups. It is interesting that the CD11c^+^CD8^+^MHCII^Low^ DC numbers were higher in mice treated with combination therapy than the OVA‐Clec9A‐TNE treatment alone group. MHCII^low^ DCs are reported to be immature DCs whose MHCII is kept low through high endocytosis and degradation in lysosomes.^16^ Likely the activation of CAROT cells and the killing of the tumour cells induced elevated levels of DC growth factors that promote the differentiation of DC precursors to immature DCs. These immature DCs were likely recruited to the inflamed tumour site and kept in an immature status alongside other immature myeloid cells within the TME.

### Functional endogenous immunity is required for combination treatment efficacy

**Figure 7:**
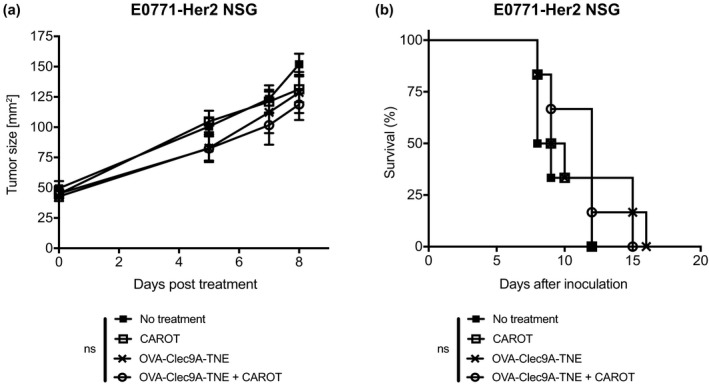
OVA‐Clec9A‐TNE and CAROT cell combination therapy efficacy requires functional endogenous immunity and DCs. E0771‐Her2 tumour‐bearing NSG mice were treated as described in Figure 4a. **(a)** Tumour growth curve of treated and untreated mice. Data are shown as mean ± SEM of *n* = 6 mice per group. Statistical significance was determined by two‐way ANOVA. **(b)** Survival curve of treated and untreated mice. Statistical significance was determined by the Mantel–Cox test (ns = not significant). Results shown are from one experiment.

We next tested the efficacy of combining OVA‐Clec9A‐TNE and CAROT cells in immunodeficient NOD.Cg‐Prkdc^scid^Il2rg^tm1Wjl^/SzJ (NSG) mice that lack functional lymphocytes and have defective DCs.^17^, ^18^ In untreated and all treatment groups, tumours grew progressively with no significant difference in tumour growth (Figure 7a). There was no significant difference in mouse survival across all the groups, and no mice survived beyond 16 days after the treatment (Figure 7b). The results indicate a requirement for functional endogenous immunity, likely antigen‐presenting cells (APCs) including cross‐presenting DCs, for the combination treatment to be effective.

In summary, combining dual‐specific CAROT cells and OVA‐Clec9A‐TNE resulted in significant tumour regression, led to long‐term survival and generated antitumour memory in immunocompetent murine tumour models. The shrinkage of tumours was accompanied by significant CD8^+^ CAROTI cell expansion and tumour infiltration. The endogenous immune system, likely the functioning of cross‐presenting DCs, was necessary for the antitumour effect.

## Discussion

While remarkably efficacious in patients with acute B‐cell leukaemia and some B lymphomas, CAR T cells have far more limited efficacy in solid tumours because of the immunosuppressive TME.^19^ In this study, we exploited a nanoparticulate vaccination platform for cross‐priming CAR T cells by cross‐presenting DCs, which are present in lymphoid organs and tumours. Here, we demonstrate that the use of dual‐specific CAR T cells, in combination with OVA‐Clec9A‐TNE, mediates durable remission of solid tumours in mice.

Clec9A is a C‐type lectin receptor that is selectively expressed by cross‐presenting DCs, in both humans and mice. We hypothesised that the OVA antigen delivered by OVA‐Clec9A‐TNE could be presented by cross‐presenting DCs to both CAROTI and CAROTII cells to induce antitumour CAR response in solid tumour models. Excitingly, the adoptive transfer of CAROT cells in combination with OVA‐Clec9A‐TNE treatment drove significant tumour regression and generated immune memory responses against tumour cells *in vivo* with no observed toxicities, indicating that this combination overcomes the limitation of effective antigen presentation for the generation of antitumour immunity. Significant CAROTI and CAROTII cell proliferative responses and CAROTI IFN‐γ secretion in response to OVA‐Clec9A‐TNE treatment were observed. This response was mediated by the OVA‐specific TCR stimulation. It will be interesting in future studies to characterise the activation and memory phenotype of both adoptively transferred CAROT and host T cells. Phenotypic analysis for memory cell markers will be helpful to evaluate the mechanisms by which CAROTI cells accumulate in tumour sites, and to determine how immune memory is generated in CAROT cells and/or the endogenous immune system.

The OVA‐Clec9A‐TNE drove CAROTI cell proliferation both *in vitro* and *in vivo*, and the OVA‐Clec9A‐TNE vaccine boosted CAROT cell antitumour immunity via OVA‐specific TCR stimulation. Interestingly, although we observed the expansion of CAROTII cells *in vitro*, we did not observe a significant increase in CAROTII cell numbers in the spleens or tumours in response to OVA‐Clec9A‐TNE treatment in tumour‐bearing mice. Targeting antigen to DCs using anti‐Clec9A was shown to elicit both CD8^+^ and CD4^+^ T‐cell proliferative responses in naïve mice and Clec9A‐OVA‐TNE expanded adoptively transferred OTII T cells in naïve mice.^7^, ^20^ It is possible that CAROTII cells downregulated CD4 expression upon persistent vaccine and tumour‐derived antigen stimulation so that the CAROTII cells were not detected by flow cytometry. Alternatively, the expanded CAROTII cells might have migrated to other sites where Clec9A‐TNE delivered the antigen, such as the liver.^7^ Nevertheless, our results demonstrated that the OVA‐Clec9A‐TNE effectively drives antigen cross‐presentation to the CD8^+^ CAROTI cells.

Although our previous study demonstrated that OVA‐Clec9A‐TNE was superior to soluble OVA protein in stimulating OTI and OTII cells *in vivo*,^7^ we did not directly compare OVA‐Clec9A‐TNE to OVA protein in stimulating CAROT cells and the antitumour effect. Usually, a strong adjuvant is required to elicit an effective antitumour T‐cell response, but it will be beneficial in future studies to include additional experimental control groups to further strengthen the conclusion.

We observed a high level of cross‐presenting DCs in the spleens of mice that received combination treatment (Figure 6e). It is likely these cross‐presenting DCs present antigen to CAROTI cells and facilitate CAROTI cell expansion. A significant level of tumour‐infiltrating DCs (TIDCs) was also observed in tumours of combination‐treated mice (Figure 6f). A variety of TIDC subsets exist, where many play a role in cancer immune‐surveillance by driving adaptive immune responses against tumour antigens.^21^ In fact, high frequencies of TIDCs correlate with favourable prognoses in a variety of cancers.^22^, ^23^ Elevated levels of TIDCs have been shown to improve CD8^+^ T‐cell persistence in tumours,^24^, ^25^ and once expanded, TIDCs can be targeted by Clec9A‐TNE. Thus, elevated TIDC and CAROTI cell persistence and repeated OVA‐Clec9A‐TNE dosing are likely to work together to promote T‐cell memory.

A combination of OVA‐Clec9A‐TNE and CAROT cell treatment did not drive significant tumour regression in the NSG mice bearing E0771‐Her2 tumours. As NSG mice do not produce functional lymphocytes and have compromised DC function,^17^, ^18^ our data suggest that endogenous DC and/or lymphocytic activity is necessary for combination treatment to mediate tumour regression. In adoptive transfer experiments in Her2 transgenic hosts, mice were preconditioned, receiving total body irradiation at a dose that depletes endogenous lymphocytes and some DCs. Clearly, DCs return over the days following irradiation, but it would be of interest to determine whether treatment efficacy can be improved by using preconditioning regimens that preserve DC populations.

It has been demonstrated that TCR signalling could abrogate the anti‐cancer effect of CD8^+^ CAR T cells but not CD4^+^ T cells through reduced expansion, increased apoptosis and exhaustion in a CD19^+^ ALL murine model. Differential gene expression profile revealed that stimulation through TCR and CAR simultaneously generated distinct patterns in CD8^+^ and CD4^+^ CAR T cells, with TCR signal more prevalent in CD8^+^ CAR T cells and CAR signal more dominant in CD4^+^ CAR T cells.^26^ It is interesting that we observed significant expansion and infiltration of CD8^+^ CAROTI cells post‐treatment with Clec9A‐OVA‐TNE, but did not modulate CD4^+^ CAROTII cells (Figure 6). Our data suggest that in our system, the engagement of TCR mainly enhanced the activation of CD8^+^ CAR T cells. The difference is likely due to the cells that provide the TCR signalling. In our study, antigen presentation was provided by the Clec9A^+^ DCs, while in the previous study, by the non‐cancer cells positive for a HY male‐specific antigen.

Enhancing CAR T‐cell antitumour effect by engaging CAR T‐cell TCR has been demonstrated before, by us^6^ and others. In our previous study, we used a vaccinia viral vaccine coding TCR peptide to stimulate the TCRs on the CAR T cells, which resulted in the eradication of large tumours and metastases in a number of murine Her2 tumour models. The TCR peptide is likely presented by APCs. Subsequently, a number of studies elegantly demonstrated that by enhancing CAR T‐cell interaction to APCs, large tumours in difficult murine models could be effectively treated.^27^, ^28^, ^29^ The cells providing TCR signals are APCs in these studies, which also provide T‐cell co‐stimulation and cytokines simultaneously. In contrast, CAR interacts with tumour cells, which usually also provide inhibition signals to the CAR T cells. Therefore, linking CAR T cells with APCs, either through the stimulation of TCR or other approaches, could be a valid strategy in enhancing CAR‐mediated tumour cell killing. How the TCR stimulation enhances CAR‐mediated activity at a cellular level warrants further investigation.

The clinical application of this approach may involve transduction of patient T cells with two gene constructs – one encoding a conventional CAR and the second, a TCR detecting a known, tumour peptide presented in MHCI. Transducing the same T cell with a CAR and a TCR has been demonstrated feasible.^30^ Currently, conventional CAR T‐cell therapy has had little success when applied to solid cancers, but we propose that incorporating both CAR T cells and simultaneous vaccination against a tumour antigen detected by the transgenic TCR presents a promising and testable avenue for improving patient outcomes in a variety of poor outcome human cancers.

## Methods

### Mice and cell lines

All mouse experiments were approved by the Peter MacCallum Cancer Centre Animal Experimentation Ethics Committee (Protocol E582). All the mice were bred at the Peter MacCallum Cancer Centre (Victoria, Australia), and mice used for experiments were between 6 and 16 weeks of age.

The parental murine breast cancer E0771‐LMC and chemically induced colon adenocarcinoma MC38 cell lines were previously transduced to stably express the full‐length human Her2 antigen under control of the mouse stem cell virus LTR promoter (MSCV) by retroviral transduction as described.^31^ Parental and Her2‐expressing cell lines were cultured in supplemented DMEM media as described previously.^6^, ^29^

### Tumour inoculation and treatment

C57BL/6‐Her2 transgenic or NSG mice were injected subcutaneously with 5 × 10^5^ E0771‐Her2 cells or MC38‐Her2 cells. Tumours were allowed to establish over 7–8 days with sizes ranging between 35 and 45 mm^2^. Mice were left untreated or irradiated (4 Gy) before treatment (day 0). Treated mice received intravenous injections of OVA‐Clec9A‐TNE (10 μg 100 μL^−1^), CAROTI (1 × 10^7^ cells 100 μL^−1^) and CAROTII splenocytes (1 × 10^7^ cells 100 μL^−1^) or a combination of both OVA‐Clec9A‐TNE, CAROTI and CAROTII splenocytes (Figure 4a). Treated mice received intraperitoneal injections of rhIL‐2 (1 × 10^5^ IU 200 μL^−1^) once on day 0, twice on day 1 and once on day 2. Mice that received the OVA‐Clec9A‐TNE also received an additional injection of TNE on day 7. Tumours were measured three times a week. Mice were culled when tumours grew up to the ethical limit of 150 mm^2^ or when showed signs of distress, including hunched/ruffled appearance or lethargy.

In the rechallenge experiment, surviving, tumour‐free mice were inoculated subcutaneously with 1 × 10^5^ E0771‐Her2 cells 60 days after the primary tumour rejection. Naïve C57BL/6‐Her2 transgenic mice were injected at the same time as the experimental controls.

### Preparation of tailored TNEs

Tailored TNE was prepared as previously described.^7^ Briefly, OVA protein (Sigma‐Aldrich, St Louis, MO, USA) was first emulsified with a lipophilic surfactant to form a water‐in‐oil (W/O) emulsion. Following removal of the water phase, a solid lipophilic antigen complex was formed and dissolved in oil to form a solid‐in‐oil emulsion (S/O). The S/O was emulsified with a peptide surfactant and the Clec9A targeting Trp‐His (WH) peptide to form the TNE. OVA carrying TNE (OVA‐Clec9A‐TNE) were prepared in 5 μg OVA 100 μL^−1^ and 10 μg OVA 100 μL^−1^ doses. An empty‐vehicle TNE was additionally prepared as vehicle control.

### T‐cell culture and cellular assays

Spleens were removed from euthanised mice, digested in 1 mg mL^−1^ collagenase III (Worthington, Lakewood, NJ, USA) and 365 U mL^−1^ of deoxyribonuclease I (DNAse I) (Thermo Fisher Scientific, Waltham, MA, USA) for 30 min at 37°C. The cells were subsequently incubated with 100 mm EDTA for additional 10 min at room temperature before being filtered through 70 μm filters to form a single cell suspension. After red blood cell lysis, the lymphocytes were resuspended in supplemented RPMI medium.

Splenocytes were resuspended to 1 × 10^6^ cells mL^−1^ in supplemented RPMI media with 100 IU mL^−1^ recombinant human (rh) IL‐2 (rhIL‐2) (Jiangsu Kingsley Pharmaceutical Co Ltd, Wuxi, China) and 5 μm SIINFEKL peptide (CAROTI) (Auspep, Tullamarine, VIC, Australia), 5 μm OVA_323‐339_ peptide (CAROTII) (Auspep) or 0.5 μg mL^−1^ of αCD3 and 0.5 μg mL^−1^ αCD28 (WT). Cells were maintained in a humidified incubator at 37°C and 5% CO_2_. Every 48 h, cells were resuspended to 1 × 10^6^ cells mL^−1^ in fresh supplemented RPMI media containing 100 IU mL^−1^ of rhIL‐2. Cells were harvested to use for cellular assays or adoptive transfer 7 days after initial culture.

To test the cytotoxicity of T cells, 5 × 10^6^ E0771‐LMC or E0771‐Her2 tumour cells were labelled with 75 μCi ^51^Chromium (Perkin Elmer, Waltham, MA, USA) and plated at 2 × 10^4^ cells per well in 96‐well U‐bottom plates. Stimulated CAROT cells were added to tumour cells at ratios of 40:1, 20:1, 10:1, 5:1 and 1:1. Appropriate cells were used as experimental controls. After 14‐h incubation, supernatant radioactivity was measured using the Wallac Wizard 1470 automatic gamma counter (Global Medical Instrumentation Inc., Ramsey, MN, USA). Percentage‐specific lysis was determined using the following formula: [(Experimental lysis − spontaneous lysis)/(Total lysis − spontaneous lysis)] × 100.

### Antibodies

Anti‐mouse CD3 (clone: 145‐2C11) and anti‐mouse CD28 (clone: 37.51) antibodies used for splenocyte stimulation were purchased from BD Biosciences (Franklin Lakes, NJ, USA). IgG2a isotype (clone: 2A3) was purchased from BioXCell (Hanover, NH, USA). Antibodies for flow cytometry and IHC are αCD4‐APC‐eF780 (clone: RM‐45), αCD8a‐BV811 (clone: 53‐6.7), αCD8 purified (clone: 4SM15), αCD11c‐BV785 (clone: N418), αCD11b‐APC‐eF780 (clone: M170), αCD45.1‐FITC (clone: A20), αClec9A‐PE (clone: 10B4), α‐c‐Myc‐AF647 (clone: 9E10), αF4/80‐eF450 (clone: BM8), αI‐A/I‐E‐APC (clone: M5/114.15.2) and αTCR Vα2‐PE‐Cy7 (clone: B20.1).

### Flow cytometry, cytokine assay and IHC

Cells for flow cytometric analysis were pelleted and stained in 50 μL per well of fluorochrome‐conjugated antibody cocktails diluted in FACS buffer (PBS containing 0.5% FCS and 2 mm EDTA) for 30 min at 4°C in the dark. Following washing and resuspension in FACS buffer, cells were labelled with 2 μm Fluorogold viability marker (Biotium, Hayward, CA, USA) and, 20 μL of Flow‐Count Fluorospheres (Beckman Coulter, Indianapolis, IN, USA) was added to each sample. Samples were acquired on the BD LSR II (BD Bioscience). Data were analysed and plotted using FlowJo v10.4 (Tree Star Inc., Ashland, OR, USA).

For serum cytokine analysis, blood from mice was collected and allowed to clot for 30 min and centrifuged for 10 min at 1000 *g*. Serum was collected and stained using the LEGENDplex mouse inflammation panel kit (BioLegend, San Diego, CA, USA) according to the manufacturer's instructions. To determine cytokine secretion from T cells *in vitro*, CAROT cells were stimulated with peptides, and the supernatant was harvested. IL‐2, IFN‐γ and TNF‐α were analysed using a BD CBA kit according to the manufacturer's instructions (BD Bioscience). Samples were analysed using the BD FACSVerse (BD Bioscience), and data were analysed using FCAP array software (BD Bioscience).

CD8 IHC was carried out as described previously.^29^ In brief, 4 μm tissue sections were stained with 5 μg mL^−1^ antibody against CD8 (clone 4SM15, eBioscience, San Diego, CA, USA). Antigen retrieval using a high temperature 10 mm citrate buffer (pH = 6) was performed before the primary antibody incubation. ImmPress horseradish peroxidase (HRP)‐conjugated anti‐rat IgG (Vector Laboratories Ltd, Burlingame, CA, USA) and 3,3′‐diaminobenzidine (DAB) substrate (Vector Laboratories Ltd) were used for colour development. Slides were imaged using an Olympus VS120 microscope (Olympus, Tokyo, Japan) and VS‐ASW software (Olympus). Images were analysed using HALO (Indica Labs, Corrales, USA) where positive staining was quantified as the number of stain positive divided by tissue area in mm^2^.

### Graphical presentation and statistical analysis

Data were analysed and graphed using GraphPad Prism 7 (GraphPad, La Jolla, CA, USA) and Microsoft Excel (Microsoft Corporation, Albuquerque, NM, USA). One‐way ANOVA was used to compare groups of data in *in vitro* studies, and Tukey's range test was used to correct for multiple comparisons. Tumour growth was compared using two‐way ANOVA, and Tukey's range test was used to correct for multiple comparisons. Mouse survival was compared using the Mantel–Cox test. Significant differences between comparisons were indicated by *P* values ≤ 0.05.

## Conflict of interest

The authors declare no conflict of interest.

## Author contributions

**Jack D Chan:** Conceptualization; Data curation; Formal analysis; Investigation; Methodology; Validation; Visualization; Writing‐original draft; Writing‐review & editing. **Bianca von Scheidt:** Investigation; Methodology; Writing‐review & editing. **Bijun Zeng:** Conceptualization; Investigation; Methodology; Writing‐review & editing. **Amanda J Oliver:** Investigation; Methodology; Writing‐review & editing. **Ashleigh S Davey:** Investigation; Methodology; Writing‐review & editing. **Aesha I Ali:** Investigation; Methodology; Writing‐review & editing. **Ranjeny Thomas:** Conceptualization; Data curation; Resources; Writing‐original draft; Writing‐review & editing. **Joseph A Trapani:** Conceptualization; Funding acquisition; Investigation; Methodology; Supervision; Writing‐review & editing. **Phillip K Darcy:** Conceptualization; Data curation; Formal analysis; Funding acquisition; Project administration; Supervision; Writing‐review & editing. **Michael H Kershaw:** Conceptualization; Data curation; Formal analysis; Funding acquisition; Investigation; Methodology; Project administration; Resources; Software; Supervision; Validation; Writing‐original draft; Writing‐review & editing. **Riccardo Dolcetti:** Conceptualization; Data curation; Formal analysis; Funding acquisition; Methodology; Project administration; Resources; Supervision; Writing‐original draft; Writing‐review & editing. **Clare Y Slaney:** Conceptualization; Data curation; Formal analysis; Funding acquisition; Investigation; Methodology; Project administration; Supervision; Writing‐original draft; Writing‐review & editing.

## Supporting information

Supplementary figure 1

Supplementary figure 2

Supplementary figure legends
