# Supplementary material for: Enhancing chimeric antigen receptor T‐cell immunotherapy against cancer using a nanoemulsion‐based vaccine targeting cross‐presenting dendritic cells
Source: Clin Transl Immunology. 2020 Jul 22;9(7):e1157. doi: 10.1002/cti2.1157 (PMC7374388; doi:10.1002/cti2.1157)
Supplement: Supplementary file 3 — Supplementary figure legends [file CTI2-9-e1157-s003.docx]

**Supplementary figure 1. E0771-Her2 cells express Her2.** Flow cytometric assay demonstrating positive Her2 staining on E0771-Her2 cells and negative Her2 staining on E0771-LMC cells. Representative data from more than three experiments.

**Supplementary figure 2.** **The majority of the CD44^Low^CD62L^High^ CAROT cells post OVA-Clec9A-TNE incubation are undivided naïve cells.** 1 x 10^6^ CAROTI splenocytes labeled with 0.1 μM CFSE were cultured over 72 hours with OVA-Clec9A-TNE at an OVA concentration of 50 nM. Following culture, CAROT cells were quantified via flow cytometric analysis. CFSE dilution was analysed on the CAR^+^TCRVα2^+^CD44^low^CD62L^High^ population. Cells cultured in the presence of 50 nM OVA protein, empty-vehicle TNE or medium only were used as the negative controls and the cells cultured with soluble αCD3 (0.5 μg mL^-1^) and αCD28 (0.5 μg mL^-1^) were used as positive controls. Representative data from more than three experiments.
